# Epidemisches Versagen: Warum Staaten und internationale Organisationen wiederholt bei der Bekämpfung von Epidemien scheitern

**DOI:** 10.1007/s12399-021-00876-3

**Published:** 2021-11-24

**Authors:** Dirk Richter, Simeon Zürcher

**Affiliations:** 1 Departement Gesundheit, Berner Fachhochschule, Murtenstrasse 10, 3008 Bern, Schweiz; 2 Zentrum Psychiatrische Rehabilitation, Universitäre Psychiatrische Dienste Bern, Bern, Schweiz

**Keywords:** Epidemie, Pandemie, Ebola, Coronavirus, Epidemic, Pandemic, Ebola, Coronavirus

## Abstract

Warum haben Staaten und internationale Organisationen wiederholt bei der Epidemiebekämpfung versagt? Wir analysieren das Vorgehen währen der Ebolaepidemie und der SARS-CoV‑2-Pandemie. In beiden Fällen erfolgte die Reaktion im Rahmen eines Zyklus epidemischen Versagens (ZEV) mit folgenden Phasen: Vernachlässigung, Arroganz/Leugnung, Panik und Analyse/Selbstkritik. Zentrale Ursachen für den ZEV sind die Ökologie (v.a. die Vernachlässigung von Zoonosen), die Politik (v.a. negative Anreize bei der Epidemiebekämpfung), sozioökonomische Kontexte, die menschliche Psychologie (v.a. kognitive Verzerrungen) sowie erkenntnistheoretische Probleme (v.a. der Rückgriff auf die Erfahrungen früherer Epidemien).

## Abstract (en)

Why have states and international organizations repeatedly failed to successfully respond to epidemics? We analyze the response during the Ebola epidemic and the SARS Cov‑2 pandemic. In both cases, the response occurred within an Epidemic Failure Cycle (EFC) with the following phases: Negligence, Arrogance/Denial, Panic, and Analysis/Self-Criticism. Central causes of ZEV include ecology (especially neglect of zoonotic diseases), politics (especially negative incentives in epidemic control), socioeconomic contexts, human psychology (especially cognitive biases), and epistemological problems (especially recourse to the experience of previous epidemics).

## Einleitung

Die Weltgemeinschaft hat innerhalb eines Jahrzehnts zweimal versagt, als es darum ging, auf größere Virusepidemien zu reagieren: bei der Ebolaepidemie im Jahr 2014 und bei der SARS-CoV‑2-Pandemie im Jahr 2020. Diese Versäumnisse traten auf, obwohl die Schwächen und die mangelnde Vorbereitung lange bekannt waren (Lakoff 2017). Darüber hinaus mangelte es nicht an Empfehlungen hinsichtlich notwendiger Maßnahmen. Viele nationale und internationale Organisationen sowie Gremien hatten Daten und Ratschläge zur Verfügung gestellt, damit sich die Weltgemeinschaft auf die nächste Pandemie, die „Krankheit X“, hätte vorbereiten können (GHS Index 2019). Die zuletzt im Jahr 2019 formulierten Vorschläge umfassen Themen wie die Stärkung der Gesundheitssysteme, die Surveillance von Ausbrüchen bei Tieren und Menschen, das Monitoring der Vorbereitung und der Biosicherheit, die Stärkung der Versorgungsketten und die Koordinierung der Initiativen (Bloom und Cadarette 2019).

Trotz dieser Bemühungen wurden die meisten Länder Anfang 2020 von der weltweiten Ausbreitung der SARS-CoV‑2-Pandemie überrascht. In vielen Ländern waren die Gesundheitssysteme vor allem im Jahr 2020 mit schweren Covid-19-Fällen überfordert, die Schutzausrüstung für das Gesundheitspersonal war knapp, die Versorgungsketten für Masken und andere Ausrüstungsgegenstände funktionierten nicht und die internationale Koordinierung wurde durch einseitige Bemühungen der Länder des globalen Nordens bezüglich des Kaufs solcher Ausrüstungsgegenstände behindert.

Offensichtlich haben viele Länder nicht aus den Fehlern der ersten Pandemiewelle gelernt und wurden von nachfolgenden Wellen heimgesucht, die sich als noch verheerender erwiesen. Zwei Fragen sind daher von Bedeutung, die wir nachfolgend zu beantworten versuchen: 1) Was läuft bei den nationalen und internationalen Reaktionen auf Epidemien wiederholt falsch, und 2) warum läuft es falsch?

## Reaktion auf Epidemien und ihr Versagen: Der Forschungsstand

Die bisherige Forschung über die Reaktion auf Epidemien und das Scheitern von Maßnahmen beschränkt sich in der Regel auf nationale Untersuchungen, sogenannte *After Action Reviews* (AAR) und/oder Berichte von internationalen Organisationen wie die Untersuchungen der Rolle der Weltgesundheitsorganisation (WHO) während der Ebolaepidemie. Die oft lokalen und nationalen Berichte, welche sich mit spezifischen Fragen einer Epidemie befassen, stellen erhebliche Herausforderungen für die Generalisierung von Erkenntnissen dar (Stoto et al. 2019).

Allgemein betrachtet, folgen Epidemien und Pandemien in der Regel einem Zyklus, der aus vier Phasen besteht. Der aktuelle WHO-Leitfaden für eine Influenzapandemie beschreibt etwa die folgenden Phasen: Pandemische Zwischenphase, Warnphase, Pandemie, Übergang und dann zurück zur Zwischenphase (WHO 2017). Weitere Zyklusansätze entsprechen ebenfalls weitgehend einem vierphasigen Rahmen. In Bezug auf HIV/AIDS postulierte der Medizinhistoriker Charles Rosenberg bereits in einer frühen Arbeit folgende Abschnitte: fortschreitende Aufdeckung, Bewältigung und Rationalisierung des Zufalls, Verhandlung der gesellschaftlichen Reaktion und einen Epilog, in dem die Lehren aus dem Ausbruch gezogen werden (Rosenberg 1989). Neuere Ansätze legen ebenfalls nahe, dass es mindestens vier Phasen zur Beschreibung von Reaktionszyklen gibt, z. B. Entdeckung, frühzeitige Reaktion, Intervention und Nachbereitung der Intervention (Polonsky et al. 2019) oder ein weiterer Ansatz, welcher die Überwachung/Vorbereitung vor der Entdeckungsphase mit einbezieht (Katz und Graeden 2020).

Was das Scheitern von Maßnahmen angeht, so ist man sich weitgehend einig, dass sowohl die nationalen als auch die internationalen Reaktionen auf Epidemien bestimmten Abläufen folgen, die zwischen dem Ausbleiben von Maßnahmen auf der einen und drastischen Aktionen auf der anderen Seite schwanken (World Bank 2017). Während des Ebola-Ausbruchs 2014 scheinen nationale und internationale Akteure einem Zyklus von Angst und Apathie gefolgt zu sein (Price-Smith und Porreca 2016). Angesichts des SARS-1-Ausbruchs 2002/2003, der innerhalb eines kurzen Zeitraums eingedämmt werden konnte, und der H1N1-Influenzapandemie 2009, die in den Augen von Politik und internationalen Organisationen relativ schwach war, sei generell von starken Gegenmaßnahmen abgesehen worden, so die Analyse. Als der Ausbruch außer Kontrolle geriet, wurde jedoch mit energischen Maßnahmen reagiert, wie auch später in diesem Beitrag gezeigt wird. Weitere stufentheoretische Darstellungen von Gesundheitskatastrophen gehen von einem Zyklus aus, der aus zwei Stufen besteht, wie z. B. Krise und Selbstzufriedenheit (CSIS 2019) oder Panik und Vernachlässigung (World Bank 2017). Die letztgenannte Terminologie wurde von einem Gremium der Weltbank zur Finanzierung der Gesundheitsvorsorge geprägt, das feststellte, dass während einer Gesundheitskatastrophe eine relevante Panik besteht und dass nach einiger Zeit die Exposition gegenüber solchen Gefahren weitgehend ignoriert wird und die Phase der Vernachlässigung eintritt. Allen diesen Ansätzen ist gemein, dass ein Nichthandeln in eine harte und drastische Reaktion umschlägt, wenn der Infektionsausbruch außer Kontrolle zu geraten scheint.

Es ist offensichtlich, dass nationale Behörden und internationale Organisationen große Schwierigkeiten haben, aus früheren Ausbrüchen und Katastrophen zu lernen (Donahue und Tuohy 2006; Segovia und Ébodé 2020). Dies bezieht sich unter anderem auf Unklarheiten bezüglich der Definition einer Epidemie und des Zeitpunkts, eine solche auszurufen (Mullen et al. 2020).

Es hat sich zudem gezeigt, dass relevante Forschungsergebnisse zu Infektionskrankheiten und Fragen der Gesundheitssicherheit oft nicht bei Personen mit politischer Entscheidungskompetenz ankommen (Berger et al. 2019).

Zusätzlich zu den festgestellten Ungereimtheiten und Wissenslücken sieht sich die Politik mit speziellen Problemen und Hindernissen bei der Epidemiebekämpfung konfrontiert. Die Reaktion auf eine Epidemie kann sich auf die lokale, nationale und internationale Politik auswirken, und die Folgen sind sehr oft in den ärmeren Sozialmilieus zu spüren (Kapiriri und Ross 2020). Daher ist es denkbar, dass die Politik ungern eine Epidemie in dem jeweiligen Land ausruft, was gemeinhin als Versagen der jeweiligen Behörden angesehen wird (Rull et al. 2015). Aus dieser Perspektive wird klarer, dass eine unzureichende politische Mobilisierung einer der Hauptgründe für Verzögerungen bei der Ausrufung eines Gesundheitsnotstands internationaler Tragweite (*Public Health Emergency of International Concern*, PHEIC) ist (Hoffman und Silverberg 2018). Zusammenfassend lässt sich sagen, dass Versäumnisse bei der Reaktion auf Epidemien mit einer inhärenten Zyklizität, Wissenslücken und Fehlanreizen für politische Entscheidungen verbunden sind.

## Der Zyklus des epidemischen Versagens

Im Folgenden entwickeln wir die Hypothese des Zyklus des epidemischen Versagens (ZEV). Die detaillierten Hintergründe der Hypothese werden im nächsten Abschnitt vorgestellt. Wir halten die Phasen der Panik und der Vernachlässigung aus dem Bericht der Weltbank zunächst für einen sehr nützlichen Ausgangspunkt. Wie oben angedeutet, sind wir jedoch der Ansicht, dass der vollständige Zyklus zusätzliche Phasen benötigt, ähnlich wie bei allgemeinen Theorien über Epidemiezyklen. Sowohl in der historischen (Honigsbaum 2020) als auch in aktuellen Literatur (Bouska 2020; Garrett 2018) darüber, wie Gesellschaften auf Epidemien reagieren, wird die Bedeutung von Arroganz oder Hybris hervorgehoben. Wir schlagen daher vor, diese Phase als erste zusätzlich in den Zyklus von Panik und Vernachlässigung aufzunehmen. Arroganz, Hybris und Verleugnung sind vor allem in der Alarm‑/Entdeckungsphase von Bedeutung, in der die Politik und die Öffentlichkeit zunehmend erkennen, dass eine große Bedrohung vorhanden ist und dass erhebliche Anpassungen erforderlich sind. Auf der anderen Seite der Panikphase sehen wir die Notwendigkeit einer zweiten Ergänzung des Zyklus aus Vernachlässigung und Panik mit einer Phase, die wir Analyse/Selbstkritik nennen. Nach großen epidemischen Ausbrüchen wird häufig untersucht, wie Organisationen und Nationalstaaten mit der Epidemie umgegangen sind.

**Figure 1:**
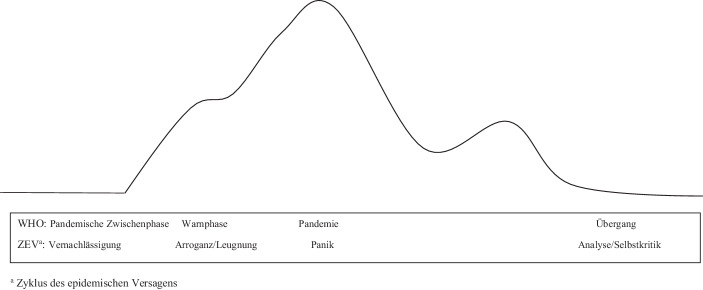

Die Hypothese des ZEV besteht demnach aus vier Phasen: Vernachlässigung, Arroganz/Leugnung, Panik und Analyse/Selbstkritik. Die ZEV-Phasen verlaufen weitgehend parallel zum WHO-Konzept der Zwischenpandemischen Phase, der Alarmphase, der Pandemie- und der Übergangsphase (Abbildung 1). Die *Phase der Vernachlässigung *bezieht sich hauptsächlich auf zwei Aspekte epidemischer Ausbrüche. Erstens werden potenzielle Quellen für virale Ausbrüche ignoriert, wie wir es bei der Bedrohung durch Zoonosen wiederholt gesehen haben. Zweitens wird die Nichtvorbereitung von Organisationen und Staaten ignoriert. Die *Phase der Arroganz/Verleugnung *zeigt sich, wenn Politik und Öffentlichkeit kommunizieren, dass – trotz steigender Infektionszahlen – alles unter Kontrolle sei und/oder dass die Bedrohung nicht existiere oder übertrieben sei. Die *Panikphase *kommt ins Spiel, wenn plötzliche dramatische Veränderungen in der Reaktion zu beobachten sind, wie beispielsweise groß angelegte Quarantäne- oder Lockdownmaßnahmen. Die *Phase der Analyse/Selbstkritik *findet zumeist statt, nachdem der Ausbruch unter Kontrolle ist, wenn die Beteiligten über ihre Rolle während der Epidemie und über die Lehren nachdenken, die für künftige Wellen oder Ausbrüche zu ziehen sind.

## Der Zyklus des epidemischen Versagens während des Ebolaausbruchs 2014 und der SARS-CoV‑2-Pandemie 2020

In diesem Abschnitt werden Einzelheiten des Ebolaausbruchs 2014 und der SARS-CoV‑2-Pandemie 2020 analysiert. Die Forschungsliteratur über die Reaktion auf den Ebola-Ausbruch stammt hauptsächlich aus anthropologischen und verwandten sozialwissenschaftlichen Veröffentlichungen (z. B. Abdullah und Rashid 2017; Richards 2016; Sabeti und Salahi 2018). Die Informationen zu SARS-CoV‑2 stammen in erster Linie aus ausführlichen journalistischen Berichten, insbesondere aus dem Vereinigten Königreich (Calvert und Arbuthnott 2021), den Vereinigten Staaten (Wright 2021), der Schweiz (Recherchedesk Tamedia 2020) und Deutschland (Gloger und Mascolo 2021; Hickmann et al. 2020), sowie aus einer soziologischen Analyse des Lockdowns durch den Erstautor des vorliegenden Beitrags (Richter 2021).

### Die Phase der Vernachlässigung

Wie die meisten großen Virusausbrüche der letzten Jahrzehnte entwickelte sich das Ebolavirus in Wildtieren (Fledermäusen) und überschritt schließlich als Zoonose die Artenbarriere. Seit seinem ersten bekannten Ausbruch im Jahr 1976 gab es bis 2013 28 weitere bekannte Ausbrüche vor allem auf dem afrikanischen Kontinent (Coltart et al. 2017). Wie aus diesen Daten ersichtlich wird, war die Gefahr des Ebolavirus den nationalen und internationalen Organisationen – im Grunde genommen – gut bekannt. Die „zugrundeliegenden Ursachen dieser Epidemien (…) werden jedoch nur selten, zumindest nicht mit der nötigen Tiefe und Ausdauer, angegangen“ (Waltner-Toews 2020, S. 144–145.). In der Ebola-Situation müssen jedoch historische, politische und wirtschaftliche Aspekte berücksichtigt werden, welche die Chancen für eine angemessene Reaktion minimiert haben. Die politische Lage in Westafrika war instabil und die Region war bereits einige Jahre vor dem Ausbruch der Krankheit von Bürgerkriegen heimgesucht worden. Die neoliberale Politik hatte die Gesundheitssysteme geschwächt (Kieh 2017). Einem scharfen Kritiker der Gesundheitspolitik afrikanischer Staaten zufolge war dies jedoch nicht nur auf fehlende Ressourcen, sondern auch auf Fehlallokationen zurückzuführen (Tomori 2015).

Im Großen und Ganzen gelten viele dieser Problemlagen auch für die SARS-CoV‑2-Pandemie. Der erste große SARS-Ausbruch in Asien fand 2002/2003 statt (Anderson et al. 2004). Laut der „Global Study of Origins of SARS-CoV‑2“ (WHO 2020) wird allgemein angenommen, dass Fledermäuse eine wichtige Rolle bei der Entstehung der jüngsten Pandemie spielen. Die Bedrohung durch Coronaviren, die von diesen Tieren stammen, war gut bekannt und wurde von chinesischen Forschenden kurz vor der Pandemie als „dringliches Problem“ bezeichnet (Fan et al. 2019). In den Phasen zwischen den Pandemien wurden solche Gefahren jedoch von der nationalen und internationalen Gesundheitspolitik weitgehend ignoriert (mit einigen Ausnahmen wie der EcoHealth Alliance (2021)). Zusätzlich zu den weitgehend übersehenen zoonotischen Gefahren hatten die meisten betroffenen Länder ihre Gesundheitssysteme und ihr öffentliches Gesundheitswesen nicht darauf vorbereitet, mit größeren Ausbrüchen fertig zu werden. In vielen Staaten des Globalen Nordens waren die vormals gut ausgestatteten Reaktionssysteme in den letzten Jahrzehnten abgebaut worden, da nur wenige Fachpersonen davon ausgingen, dass sie benötigt werden würden (Hickmann et al. 2020). Darüber hinaus konzentrierte sich die Pandemieplanung fast ausschließlich auf den Ausbruch der Influenza, da dies die wahrscheinlichste pandemische Infektion zu sein schien. Influenzaausbrüche weisen jedoch andere Merkmale auf als die SARS-CoV‑2-Pandemie (Mackenzie 2020). Erkenntnistheoretisch hat dieses Vertrauen auf das Wissen über die Influenza zu Verwirrung und Verzögerungen im weiteren Verlauf der Pandemie geführt.

### Die Phase der Arroganz/Verweigerung

Aus offenbar politischen Gründen reagierten die Regierungen der drei am stärksten betroffenen Länder während des Ebolaausbruchs, Liberia, Guinea und Sierra Leone, Anfang 2014 nur sehr verhalten auf die Epidemie (Richards 2016, S. 40–41). „Die Regierungen der betroffenen Länder leugneten zunächst das Auftreten der Krankheit“ (Tomori 2014) Um den internationalen Handel und die Reiseunternehmen zu beruhigen, gab der damalige Präsident von Guinea an, der Ausbruch sei unter Kontrolle (Barry 2017, S. 74). Skepsis, Misstrauen und Leugnung in der Öffentlichkeit waren in der Region weit verbreitet (Richards et al. 2019). Auch bei internationalen Organisationen wie der WHO herrschte die Auffassung vor, dass der Ausbruch unter Kontrolle sei. Während die Hilfsorganisationen vor Ort versuchten, Gegenmaßnahmen zu ergreifen, waren sowohl die WHO als auch die US-Zentren für Seuchenkontrolle (CDC) davon überzeugt, dass die Situation lokal bewältigt werden konnte (Garrett 2015). Der Hauptgrund für diese Fehleinschätzung, welche zu Tausenden von Todesfällen führte, lag in der Überzeugung, dass die Epidemie sich selbst begrenzen würde, wie es bei früheren Ausbrüchen der Fall gewesen war (Honigsbaum 2017). Anders als zuvor hatte sich jedoch die Mobilität der Bevölkerung massiv verändert, so dass ländliche und städtische Regionen durch die zunehmende Motorisierung enger miteinander verbunden waren (Richards 2016, S. 48). Innenpolitische Konflikte erschwerten es zusätzlich, eine breite Akzeptanz für die notwendigen Gegenmaßnahmen zu erreichen (Barry 2017, S. 74).

Im Januar 2020 waren die meisten Regierungen und Gesundheitsbehörden außerhalb Asiens nicht besorgt über das neu aufgetretene Coronavirus. Zum einen ging man davon aus, dass die chinesische Regierung ihre Lehren aus dem SARS-CoV-Ausbruch 2002/2003 und aus weiteren kleineren Ausbrüchen gezogen hatte, und zum anderen glaubten viele Fachpersonen in den öffentlichen Gesundheitssystemen zu wissen, wie man mit SARS und ähnlichen Epidemien umgeht (Hickmann et al. 2020; Recherchedesk Tamedia 2020). In einigen Ländern führte diese Zuversicht sogar zu der Überlegung, eine Herdenimmunität zu erreichen, indem die gesamte Bevölkerung infiziert werden sollte (Calvert und Arbuthnott 2021, S. 167–193). Ähnlich wie die Regierungen während der frühen Ebolaepidemie versicherte die Politik rund um den Globus, vom deutschen Gesundheitsminister (der um „aufmerksame Gelassenheit“ bat (Hickmann et al. 2020, S. 46)) bis zum amerikanischen Präsidenten, dass alles unter Kontrolle sei. Dies war insbesondere vor Wahlen der Fall, wie empirische Untersuchungen gezeigt haben (Pulejo und Querubín 2020). Aber nicht nur die Politik war davon überzeugt, dass sie den Ausbruch der Krankheit unter Kontrolle hatte. In vielen Ländern vermittelten auch Forschende und Fachleute die gleiche Botschaft (für die USA siehe Wright (2021), für das Vereinigte Königreich siehe Calvert und Arbuthnott (2021, S. 91), für Deutschland siehe Gloger und Mascolo (2021, S. 131)). Leugnung und Verschwörungstheorien über den Ursprung des Virus waren weit verbreitet (Richter 2021).

Arroganz und Leugnung traten jedoch nicht nur in der Anfangsphase der Coronavirus-Pandemie auf. Ähnliche Haltungen waren zu beobachten, bevor in einigen Ländern Panikmaßnahmen ergriffen werden mussten, um die zweite und sogar die dritte Welle zu stoppen. Immer wieder versuchte die Politik, die Öffentlichkeit davon zu überzeugen, dass die Dinge unter Kontrolle seien und dass weitere Wellen unwahrscheinlich seien. Besonders eindrücklich war dies in Indien, wo „sich die Regierung wiederholt damit brüstete, dass die Ergebnisse serologischer Untersuchungen und des wichtigsten indischen Computermodells zur Vorhersage der Krankheitsausbreitung zeigten, dass sich das Land im ‚Endspiel‘ der Pandemie befinde“ (Padma 2021). Insgesamt, so die Schlussfolgerung des von der WHO initiierten Independent Panel for Pandemic Preparedness and Response, „hatten die Länder mit den schlechtesten Ergebnissen bei der Bekämpfung von COVID-19 unkoordinierte Ansätze, welche die Wissenschaft abwerteten, die potenziellen Auswirkungen der Pandemie leugneten, umfassende Maßnahmen verzögerten und zuließen, dass Misstrauen die Bemühungen untergrub.“ (The Independent Panel for Pandemic Preparedness and Response 2021, S. 33)

### Die Panikphase

In der zweiten Hälfte des Jahres 2014 wurde immer deutlicher, dass die Ebolaepidemie nicht mehr unter Kontrolle war. Die gesamte Epidemie war viel weiter verbreitet als frühere Ausbrüche, städtische Gebiete waren betroffen, und die Reaktionsteams stießen auf viel mehr Widerstand in der Bevölkerung (zu den Unterschieden zwischen 2014 und früheren Ausbrüchen, siehe: National Academies of Sciences, Engineering, and Medine (2016)). Die nationalen und internationalen Reaktionssysteme arbeiteten nun aktiver und mehrere Tausend internationale Mitarbeitende des Gesundheitswesens und Helfende wurden in die westafrikanische Region entsandt. Die Vereinten Nationen führten die UN-Mission für Ebola-Notfallmaßnahmen (UNMEER) durch und die Wirtschaftsgemeinschaft Westafrikanischer Staaten (ECOWAS) finanzierte Hilfsmissionen (Rashid 2017). Vor Ort wurden Ebola-Behandlungszentren eingerichtet, die Rückverfolgung von Kontaktpersonen wurde eingeführt und die Zahl der Labors wurde erhöht (Sabeti und Salahi 2018).

Im zweiten Quartal 2020 hatten viele Länder Gegenmaßnahmen gegen die SARS-CoV‑2-Pandemie ergriffen, die bis dahin unbekannt waren. Nach der chinesischen und der italienischen Regierung wurden in mehreren Ländern Lockdownmaßnahmen mit groß angelegten Quarantänen und Ausgangssperren eingeführt, während andere Länder mit geringeren Erkrankungsraten lediglich den nationalen und internationalen Reiseverkehr aussetzten, Geschäfte und Veranstaltungsorte schlossen und Bürgerinnen und Bürger dazu verpflichteten, wenn möglich von zu Hause aus zu arbeiten. Behandlungseinrichtungen wurden aufgestockt, Test- und Rückverfolgungsprogramme eingerichtet und in vielen, aber nicht allen Nationalstaaten wurden Abstandsregelungen und das Tragen von Gesichtsmasken vorgeschrieben (Richter 2021).

### Die Phase der Analyse/Selbstkritik

Die westafrikanische Ebolaepidemie löste eine beträchtliche Anzahl von nationalen und internationalen Berichten aus. Bereits 2015 veröffentlichte die WHO einen „Ebola Interim Assessment Panel Report“, in dem sie einräumte, dass frühe Warnungen vor dem Ausbruch „nicht zu einer effektiven und angemessenen Reaktion geführt haben“ (WHO 2015, S. 12). Dem Bericht zufolge handelten große Teile der Organisation zu langsam und es gab viele Hindernisse für eine effektive Kommunikation innerhalb der WHO und mit ihren Mitgliedstaaten. Neben vielen weiteren Berichten stufte ein vom UN-Generalsekretär in Auftrag gegebener „Report of the High-Level Panel on the Global Response to Health Crises“ den Ebola-Ausbruch als „vermeidbare Tragödie“ ein. Der Bericht kam zu dem Schluss, dass „die WHO und andere Organisationen das Ausmaß der Bedrohung falsch eingeschätzt haben und ihre anfängliche Reaktion weitgehend unzureichend war“ (UN 2016). Ein unabhängiges Gremium kam 2015 zu dem Schluss, dass der Ebolaausbruch „systemische Schwäche“ aufgedeckt habe, und gab zehn wichtige Empfehlungen für strukturelle Reformen der globalen und nationalen Programme zur Bekämpfung der Epidemie ab (Moon et al. 2015).

Als die erste SARS-CoV‑2-Pandemiewelle in Europa eine rückläufige Tendenz der Infektionen zeigte, räumten mehrere Politiker ein, dass sie sich eine Situation wie Anfang 2020 niemals hätten vorstellen können (Richter 2021, S. 104). Sie hielten es für unwahrscheinlich, dass die Epidemie europäische Länder – wenn überhaupt – in so kurzer Zeit erreichen könnte (Recherchedesk Tamedia 2020, S. 27). In einer detaillierten Analyse der Schweizerischen Reaktion auf die erste Pandemiewelle listet ein Bericht des Zentrums für Sicherheitsstudien der ETH Zürich mehrere Punkte auf, die während der ersten Welle als unzureichend angesehen wurden (Thränert und Zogg 2020). Die Schlussfolgerung „zu wenig, zu spät“, die auch nach der Ebolapandemie zu hören war, wurde ebenfalls in diesem Bericht als einer der Hauptpunkte genannt. Die Schweiz und viele andere europäische Länder, so der Bericht, hätten im Februar 2020 durch Untätigkeit viel Zeit vergeudet. Neben organisatorischen Schwächen stellte der Bericht fest, dass der nationale Pandemieplan unzureichend war, weil er sich auf einen Influenzaausbruch konzentrierte, bei dem andere Bedingungen gegolten hätten. Das von der WHO initiierte Independent Panel kam zu dem Schluss, dass es „zu vielen nationalen Regierungen an soliden Vorbereitungsplänen, Kernkapazitäten im Bereich der öffentlichen Gesundheit und einer organisierten sektorenübergreifenden Koordinierung mit klarem Engagement der höchsten nationalen Führungsebene mangelte“ (The Independent Panel for Pandemic Preparedness and Response 2021, S. 18).

## Mögliche Gründe für das Versagen bei der Reaktion auf Epidemien und Pandemien

Die ZEV-Hypothese zielt auf ein besseres Verständnis der fehlgeschlagenen Reaktionen auf Epidemien und Pandemien ab. Die naheliegende nächste Frage lautet: Warum ist es während der Ebola- und der Coronavirus-Krise zu solch inadäquaten Reaktionen gekommen? Die Antwort auf die „Warum-Frage“ ist natürlich ein komplexes Thema mit vielen miteinander verknüpften wissenschaftlichen, sozialen, psychologischen und politischen Aspekten. Unserer Ansicht nach sind in erster Linie fünf Hauptbereiche für die Beantwortung dieser Frage von Bedeutung: Ökologie, Politik, sozioökonomische Bedingungen, menschliche Psychologie und Erkenntnistheorie.

### Ökologie

In der Beschreibung der ZEV-Vernachlässigungssphase wurde bereits darauf hingewiesen, dass Zoonosen als Hauptursache für Virusepidemien von globalen und nationalen Entscheidungstragenden weitgehend vernachlässigt wurden (Morse et al. 2012). Dies gilt insbesondere, wenn man die mit Zoonosen verbundene Gefahr mit der anderen aktuellen Hauptbedrohung für das Leben und die Lebensgrundlagen der Menschen, dem Klimawandel, vergleicht. Das Thema Zoonosen hat es weder auf die Tagesordnung der Weltpolitik geschafft, noch hat es die Aufmerksamkeit der Medien erhalten oder die Mittel zugesprochen bekommen, welche die Forschung zum Klimawandel zu Recht erhalten hat. Ähnlich wie der Klimawandel sind auch Zoonosen zu einem großen Teil vom Menschen verursacht. Das Eindringen in die Lebensräume von Wildtieren, der Umgang mit und der Handel mit Wildtieren, die Abholzung von Wäldern, die Intensivierung der Landwirtschaft und sogar der Klimawandel selbst sind einige der relevanten Faktoren für Zoonosen (Cascio et al. 2011; McMahon et al. 2018). Das ökologische Versagen im Zusammenhang mit Virusepidemien muss genauso betrachtet werden wie andere ökologische Versäumnisse der globalen Gemeinschaft.

### Politik

In beiden Situationen sträubten sich Politik und große Teile der Öffentlichkeit in der Phase der Arroganz/Verleugnung, das potenzielle Ausmaß des Ausbruchs anzuerkennen. Während der Ebolaepidemie stellten Forschende ein weit verbreitetes Unverständnis und Ablehnung in der guineischen Öffentlichkeit fest (Barry 2017, S. 72–77). Wie nachfolgend dargelegt wird, kann dies bis zu einem gewissen Grad auf die menschliche Psychologie zurückgeführt werden. Bei Politikerinnen und Politikern ist jedoch unklar, was wirklich geglaubt wird und was der Rhetorik dient, wenn die Bedrohung durch eine Epidemie geleugnet wird. Bei der Coronavirus-Pandemie gibt es allerdings viele Hinweise darauf, dass sowohl die für das öffentliche Gesundheitswesen Verantwortlichen als auch die politischen Entscheidungstragenden tatsächlich glaubten, den Ausbruch eindämmen zu können (Gloger und Mascolo 2021; Recherchedesk Tamedia 2020; Wright 2021). Hinzu kommt, dass die Öffentlichkeit in den nichtasiatischen Ländern in den ersten Tagen nicht bereit war, strenge Beschränkungen zu akzeptieren. Politische Konflikte erschwerten es offensichtlich zusätzlich, die Botschaft zu vermitteln. Dies wurde in den Vereinigten Staaten im Jahr 2020 mehr als deutlich, als sogar das Tragen von Masken zu einem politischen Thema wurde (Wright 2021). Ähnliches war auch im Rahmen der Ebolaepidemie der Fall: Für einige Beobachtende in Guinea hatte der Ebolaausbruch „eine ethnopolitische Dimension“ (Barry 2017, S. 75). Darüber hinaus war sowohl 2014 als auch 2020 Misstrauen zwischen den Bürgern und den Regierungen bzw. den öffentlichen Gesundheitsverwaltungen festzustellen.

### Sozioökonomische Bedingungen

Obwohl das Ebolavirus und SARS-CoV‑2 in biologischer Hinsicht völlig verschieden sind und obwohl die besonderen sozialen Situationen, in denen die Epidemie und die Pandemie die Gesellschaften getroffen haben, unterschiedlich sind, lassen sich einige auffällige sozioökonomische Gemeinsamkeiten feststellen. Vor allem muss der wirtschaftliche Druck auf einige Bevölkerungsgruppen beachtet werden. Es wird angenommen, dass der Verzehr von Wildtierfleisch (*bushmeat*) mit dem Ausbruch der Ebola-Infektion in Verbindung steht. Aufgrund wirtschaftlicher Not und lokaler Gewohnheiten ist es wahrscheinlich, dass eher benachteiligte Bevölkerungsgruppen Wildtiere jagen, handeln und verzehren (Ordaz-Nemeth et al. 2017; Schulte-Herbruggen et al. 2013). Während der SARS-CoV‑2-Pandemie wurde deutlich, dass einige der besonderen mit wirtschaftlichem Druck verbundenen Arbeits- und Lebensbedingungen, etwa in der Fleischindustrie oder in Pflegeheimen, eine Virusübertragung erleichtern (Richter 2021).

### Menschliche Psychologie

Für Laien und nicht spezialisierte Entscheidungstragende ist eine Epidemie sehr schwer zu begreifen. Das Unverständnis während des Ebolaausbruchs wurde bereits erwähnt. Falsche Vorstellungen über das exponentielle Wachstum sind ein langjähriges Forschungsthema in der Psychologie (Wagenaar und Sagaria 1975), und diese falschen Vorstellungen waren während der Coronavirus-Pandemie weit verbreitet (Lammers et al. 2020). Darüber hinaus behindern mehrere kognitive Verzerrungen eine angemessene Risikowahrnehmung und anschließende Präventionsmaßnahmen. Der Optimismus-Bias („Es wird mich nicht treffen“) ist in diesem Zusammenhang einer der bekanntesten (Bottemanne et al. 2020; Pascual-Leone et al. 2021). Von anderen Gesundheitskatastrophen wissen wir, dass weitere Verzerrungen wie „Trägheit“ oder „Vereinfachung“ starke Faktoren sind, die es schwierig machen, die notwendigen Anpassungen an eine epidemische Bedrohung vorzunehmen (Meyer und Kunreuther 2017).

### Erkenntnistheorie

Ein weitgehend übersehener Faktor für verzögerte und/oder unzureichende Reaktionen auf epidemische Bedrohungen ist die Anwendung von Erkenntnissen aus früheren Ausbrüchen auf neue Epidemien und Pandemien. Bei beiden Krisen waren Fachpersonen des öffentlichen Gesundheitswesens, der Politik und sogar der Wissenschaft von der Neuartigkeit der sozialen Situation oder der biologischen Merkmale des betreffenden Virus überrascht. Fachpersonen aus Medizingeschichte und Epidemiologie betonen jedoch gleichermaßen, nicht einfach die Geschichte der Epidemien für die Erstellung von Leitlinien für neue epidemische Situationen zu nutzen (de Waal 2020; Kucharski 2020). Während sich bei der Ebolaepidemie das soziale System seit dem letzten Ausbruch grundlegend verändert hatte, lagen die Fachpersonen bei der SARS-CoV‑2-Pandemie in mindestens zwei wichtigen Punkten falsch, als sie sich auf frühere Erfahrungen beriefen: Es handelte sich nicht um eine Influenzapandemie, wie überwiegend erwartet wurde, und im Gegensatz zum früheren SARS-Coronavirus war die Übertragung durch asymptomatische Personen weit verbreitet.

## Schlussfolgerungen

Die Hypothese des Zyklus des epidemischen Versagens liefert einige Hinweise für die Analyse unzureichender Reaktionen auf epidemische Bedrohungen. Wir schlagen vor, den ZEV als eine idealtypische Abfolge von Phasen zu sehen, die gesellschaftliche Dynamiken beschreibt, welche in jeder modernen Gesellschaft auftreten können und die nicht kulturgebunden sind. Obwohl einzelne Verwaltungen und Behörden möglicherweise nicht in jedem Fall angemessen gehandelt haben und obwohl nationale Untersuchungen hilfreich sein können, um diese Probleme aufzudecken, betrachten wir diese Probleme weitgehend als systemisch. Aus sozialwissenschaftlicher Sicht ist es erstaunlich, dass nur sehr wenige Länder in der Lage waren, angemessen auf die jüngsten großflächigen Ausbrüche von Virusinfektionen zu reagieren. Auffallend ist auch, dass unzureichendes Lernen und unzureichende Vorbereitung nicht auf Epidemien beschränkt sind, sondern ebenfalls mit anderen Gesundheitsgefahren zu identifizieren sind (Donahue und Tuohy 2006; Lakoff 2017). Dies deutet darauf hin, dass eine Dynamik im Spiel ist, die über einzelne Nationalstaaten, Organisationen und sogar Epidemien hinausgeht.

Ein einfacher *Lessons Learned*-Ansatz wird daher möglicherweise keine Lösung für das Problem wiederholter Fehlschläge nach der Coronavirus-Pandemie sein. Der Ansatz der „gelernten Lektionen“ hat in der Vergangenheit zu oft nicht zu nachhaltigen Verbesserungen geführt. In naher Zukunft wird es wahrscheinlich politische und wissenschaftliche Initiativen geben, welche in die richtige Richtung gehen. Wir sehen allerdings das erhebliche Risiko, dass aufgrund psychologischer Verzerrungen und veränderter politischer sowie finanzieller Prioritäten schlussendlich die Nachlässigkeit wieder die Oberhand gewinnt. Da niemand weiß, wann die nächste Pandemie kommt, wird sehr wahrscheinlich kurzfristigen Zielen der Vorrang eingeräumt. Die Überschrift eines Papiers eines internationalen Expertisegremiums nach dem Ebolaausbruch ist in dieser Hinsicht eine deutliche Warnung: „Post-Ebola-Reformen: ausgiebige Analysen, unzureichende Maßnahmen“ (Moon et al. 2017).

Was kann man also aus Katastrophen wie einer Epidemie lernen, die N = 1 Ereignissen ähneln? In der Soziologie ist bereits früher die Notwendigkeit einer unspezifischen Vorbereitung auf Epidemien und andere Gesundheitskatastrophen betont worden, die nicht auf besonderen Risikobewertungen und früheren Ereignissen beruht. Letzteres hatte sich sowohl beim Ebolaausbruch als auch bei der Coronavirus-Pandemie als trügerisch erwiesen (Lakoff 2017; Richter 2021). Ganz allgemein schlagen wir vor, dass Entscheidungstragende durch die Anerkennung der Gefahr der Wiederholung von epidemischem Versagen vorsichtiger werden sollten, insbesondere in den Phasen der Vernachlässigung und Arroganz. Darüber hinaus sehen wir die Möglichkeit, dass der ZEV als Hypothese für die empirische Forschung dienen kann, um herauszufinden, welche Faktoren das epidemische Versagen unterstützen oder – in positiven Fällen – verhindern.

## References

[CR1] Abdullah I, Rashid I (2017). Understanding West Africa’s Ebola epidemic: Towards a political economy.

[CR2] Anderson RM, Fraser C, Ghani AC, Donnelly CA, Riley S, Ferguson NM, Hedley AJ (2004). Epidemiology, transmission dynamics and control of SARS: The 2002–2003 epidemic. Philosophical Transactions of the Royal Society B: Biological Sciences.

[CR3] Barry AAB, Abdullah I, Rashid I (2017). Interpreting the health, social, and political dimensions of the Ebola crisis in Guinea. Understanding West Africa’s Ebola epidemic: Towards a political economy.

[CR4] Berger KM, Wood JLN, Jenkins B, Olsen J, Morse SS, Gresham L, Root JJ, Fush M, Pigott D, Winkleman T, Mppre M, Gillespie TR, Nuzzo JB, Han BA, Olinger P, Karesh WB, Mills JN, Annelli JF, Barnebei J, Lucey D, Hayman DTS (2019). Policy and science for global health security: Shaping the course of international health. Tropical Medicine and Infectious Diseases.

[CR5] Bloom DE, Cadarette D (2019). Infectious disease threats in the twenty-first century: Strengthening the global response. Frontiers in Immunology.

[CR6] Bottemanne H, Morlaas O, Fossati P, Schmidt L (2020). Does the Coronavirus epidemic take advantage of human optimism bias?. Frontiers in Psychology.

[CR7] Bouska, J. (2020). How Europe and America blew it on the pandemic: A tale of blindness and arrogance. https://www.salon.com/2020/10/22/how-europe-and-america-blew-it-on-the-pandemic-a-tale-of-blindness-and-arrogance/. Zugegriffen: 14. Febr. 2021.

[CR8] Calvert J, Arbuthnott G (2021). Failures of state: The inside itory of britain’s battle with Coronavirus.

[CR9] Cascio A, Bosilkovski M, Rodriguez-Morales AJ, Pappas G (2011). The socio-ecology of zoonotic infections. Clinical Microbiology and Infection.

[CR10] Coltart CE, Lindsey B, Ghinai I, Johnson AM, Heymann DL (2017). The Ebola outbreak, 2013–2016: Old lessons for new epidemics. Philosophical Transactions of the Royal Society B: Biological Sciences.

[CR11] CSIS – Center for Strategic and International Studies (2019). Ending the cycle of crisis and complacency in U.S. global health security – A report of the CSIS Commission on Strengthening America’s Health Security. https://www.csis.org/analysis/ending-cycle-crisis-and-complacency-us-global-health-security. Zugegriffen: 14. Sept. 2021.

[CR13] Donahue AK, Tuohy RV (2006). Lessons we don’t learn: A study of the lessons of disasters, why we repeat them and how we can learn them. Homeland Security Affairs.

[CR14] EcoHealth Alliance (2021). EcoHealth Alliance. https://www.ecohealthalliance.org/. Zugegriffen: 28. Febr. 2021.

[CR15] Fan Y, Zhao K, Shi ZL, Zhou P (2019). Bat Coronaviruses in China. Viruses.

[CR16] Garrett L (2015). Ebola’s lessons: How the WHO mishandled the crisis. Foreign Affairs.

[CR17] Garrett L (2018). Human arrogance and epidemics. Lancet.

[CR19] Gloger K, Mascolo G (2021). Ausbruch: Innenansichten einer Pandemie.

[CR20] Hickmann C, Knobbe M, Medick V (2020). Lockdown: Wie Deutschland in der Coronakrise der Katastrophe knapp entkam.

[CR21] Hoffman SJ, Silverberg SL (2018). Delays in global disease outbreak responses: Lessons from H1N1, Ebola, and Zika. American Journal of Public Health.

[CR22] Honigsbaum M (2017). Between securitisation and neglect: Managing Ebola at the borders of global health. Medical history.

[CR23] Honigsbaum M (2020). The pandemic century: One hundred years of panic, hysteria, and hubris.

[CR18] GHS Index (2019). Global Health Security Index 2019. https://www.ghsindex.org/wp-content/uploads/2020/04/2019-Global-Health-Security-Index.pdf. Zugegriffen: 14. Juli 2020.

[CR24] Kapiriri L, Ross A (2020). The politics of disease epidemics: A comparative analysis of the SARS, Zika, and Ebola outbreaks. Global Social Welfare.

[CR25] Katz, R., & Graeden, E. (2020). Outbreak Activity Library: An online, user-friendly compilation of activities essential for effective outbreak response. https://goal.ghscosting.org/export/Research%20Brief%202020-05%20-%20Essential%20activities%20for%20outbreak%20response.pdf. Zugegriffen: 12. Mai 2021.

[CR26] Kieh GKJ, Abdullah I, Rashid I (2017). The political economy of the Ebola epidemic in Liberia. Understanding West Africa’s Ebola epidemic.

[CR27] Kucharski A (2020). The rules of contagion: Why things spread - and why they stop.

[CR28] Lakoff A (2017). Unprepared: Global health in a time of emergency.

[CR29] Lammers J, Crusius J, Gast A (2020). Correcting misperceptions of exponential coronavirus growth increases support for social distancing. Proceedings of the National Academy of Sciences USA.

[CR30] Mackenzie D (2020). COVID-19: The pandemic that never should have happened, and how to stop the next one.

[CR31] McMahon BJ, Morand S, Gray JS (2018). Ecosystem change and zoonoses in the Anthropocene. Zoonoses and Public Health.

[CR32] Meyer R, Kunreuther H (2017). The Ostrich Paradox: Why we underprepare for disasters.

[CR34] Moon S, Sridhar D, Pate MA, Jha AK, Clinton C, Delaunay S, Edwin V, Fallah M, Fidler DP, Garrett L, Goosby E, Gostin LO, Heymann DL, Lee K, Leung ML, Morrison JS, Saavedra J, Tanner M, Leigh JA, Hawkins B, Woskie LR, Piot P (2015). Will Ebola change the game? Ten essential reforms before the next pandemic. The report of the Harvard-LSHTM Independent Panel on the Global Response to Ebola. Lancet.

[CR33] Moon S, Leigh J, Woskie L, Checchi F, Dzau V, Fallah M, Fitzgerald G, Garrett L, Gostein L, Heymann DL, Katz R, Kickbusch I, Morrison JS, Piot P, Sands P, Sridhar D, Jha AK (2017). Post-Ebola reforms: Ample analysis, inadequate action. British Medical Journal.

[CR35] Morse SS, Mazet JA, Woolhouse M, Parrish CR, Carroll D, Karesh WB, Lipkin WI, Daszak P (2012). Prediction and prevention of the next pandemic zoonosis. Lancet.

[CR36] Mullen L, Potter C, Gostin LO, Cicero A, Nuzzo JB (2020). An analysis of international health regulations emergency committees and public health emergency of international concern designations. BMJ Glob Health.

[CR37] National Academies of Sciences, Engeneering, and Medicine (2016). The Ebola epidemic in West Africa: Proceedings of a&nbsp;workshop.

[CR38] Ordaz-Nemeth I, Arandjelovic M, Boesch L, Gatiso T, Grimes T, Kuehl HS, Lormie M, Stephens C, Tweh C, Junker J (2017). The socio-economic drivers of bushmeat consumption during the West African Ebola crisis. PLoS Neglected Tropical Disease.

[CR39] Padma TV (2021). Indian government should heed its scientists on COVID. Nature.

[CR40] Pascual-Leone A, Cattaneo G, Macia D, Solana J, Tormos JM, Bartres-Faz D (2021). Beware of optimism bias in the context of the COVID-19 pandemic. Annals of Neurology.

[CR41] Polonsky JA, Baidjoe A, Kamvar ZN, Cori A, Durski K, Edmunds WJ, Eggo RM, Funk S, Kaiser L, Keating P, le Polain de Waroux P, Marks M, Morage P, Morgan O, Nouvellet P, Ratnayake R, Roberts CH, Withworth J, Jombart T (2019). Outbreak analytics: A developing data science for informing the response to emerging pathogens. Philosophical Transactions of the Royal Society B: Biological Sciences.

[CR42] Price-Smith A, Porreca J (2016). Fear, apathy, and the Ebola crisis (2014–15): Psychology and problems of global health governance. Global Health Governance.

[CR43] Pulejo, M., & Querubín, P. (2020). Electoral concerns reduce restrictive measures during the COVID-19 pandemic. NBER Working Paper 27498. https://www.nber.org/papers/w27498. Zugegriffen: 12. Mai 2021. 10.1016/j.jpubeco.2021.104387 PMC7980214 33776156

[CR44] Rashid I, Abdullah I, Rashid I (2017). UNMEER and the international response to the Ebola epidemic. Understanding West Africa’s Ebola epidemic: Towards a political economy.

[CR45] Recherchedesk Tamedia (2020). Lockdown: Wie Corona die Schweiz zum Stillstand brachte.

[CR46] Richards P (2016). Ebola: How people’s science helped end an epidemic.

[CR47] Richards P, Mokuwa E, Welmers P, Maat H, Beisel U (2019). Trust, and distrust, of Ebola Treatment Centers: A case-study from Sierra Leone. PLoS One.

[CR48] Richter D (2021). War der Coronavirus-Lockdown notwendig? Versuch einer wissenschaftlichen Antwort.

[CR49] Rosenberg CE (1989). What is an epidemic? AIDS in historical perspective. Daedalus.

[CR50] Rull M, Kickbusch I, Lauer H (2015). Policy debate – International responses to global epidemics: Ebola and beyond. Revue internationale de politique de développement.

[CR51] Sabeti P, Salahi S (2018). Outbreak culture: The Ebola crisis and the next epidemic.

[CR52] Schulte-Herbruggen B, Cowlishaw G, Homewood K, Rowcliffe JM (2013). The importance of bushmeat in the livelihoods of West African cash-crop farmers living in a faunally-depleted landscape. PLoS One.

[CR53] Segovia, O. G., & Ébodé, A. (2020). A comparison of the institutional management of the H1N1 influenza pandemic and the Ebola virus disease epidemic in West Africa – Why we have not learned the lesson when preparing and responding to Public Health Emergency of International Concern? *Face à Face. Regards sur la santé*. https://journals.openedition.org/faceaface/1547. Zugegriffen: 7. Apr. 2021.

[CR54] Stoto MA, Nelson C, Piltch-Loeb R, Mayigane LN, Copper F, Chungong S (2019). Getting the most from after action reviews to improve global health security. Global Health.

[CR55] The Independent Panel for Pandemic Preparedness and Response (2021). Covid-19: Make it the last pandemic. https://theindependentpanel.org/mainreport/. Zugegriffen: 12. Mai 2021.

[CR56] Thränert O, Zogg B (2020). Bulletin 2020 zur Schweizerischen Sicherheitspolitik.

[CR57] Tomori O (2014). Ebola in an unprepared Africa. British Medical Journal.

[CR58] Tomori O (2015). Will Africa’s future epidemic ride on forgotten lessons from the Ebola epidemic?. BMC Medicine.

[CR59] UN – United Nations (2016). Protecting humanity from future health crises: report of the high-level panel on the global response to health crises.

[CR12] de Waal, A. (2020). New pathogen, old politics. http://bostonreview.net/science-nature/alex-de-waal-new-pathogen-old-politics. Zugegriffen: 22. Febr. 2021.

[CR60] Wagenaar WA, Sagaria SD (1975). Misperception of exponential growth. Perception in Psychophysics.

[CR61] Waltner-Toews D (2020). On pandemics - Deadly diseases from bubonic plagues to Coronavirus.

[CR62] WHO (2015). Report of the Ebola Interim Assessment Panel. https://www.who.int/csr/resources/publications/ebola/ebola-panel-report/en/. Zugegriffen: 17. Febr. 2021.

[CR63] WHO (2017). Pandemic Influenza risk management: A WHO guide to inform and harmonize national and international pandemic preparedness and response.

[CR64] WHO (2020). WHO-convened global study of the origins of SARS-CoV‑2: China part. https://www.who.int/publications/i/item/who-convened-global-study-of-origins-of-sars-cov-2-china-part. Zugegriffen: 16. Apr. 2021.

[CR65] World Bank (2017). From panic and neglect to investing in health security: Financing pandemic preparedness at a national level. https://www.worldbank.org/en/topic/pandemics/publication/from-panic-neglect-to-investing-in-health-security-financing-pandemic-preparedness-at-a-national-level. Zugegriffen: 12. Mai 2021.

[CR66] Wright, L. (2021). The plague year: The mistakes and the struggles behind America’s Coronavirus tragedy. The New Yorker. https://www.newyorker.com/magazine/2021/01/04/the-plague-year. Zugegriffen: 6. Apr. 2021.

